# Crustal velocity and interseismic strain-rate on possible zones for large earthquakes in the Garhwal–Kumaun Himalaya

**DOI:** 10.1038/s41598-021-00484-3

**Published:** 2021-10-28

**Authors:** John P. Pappachen, Rajesh Sathiyaseelan, Param K. Gautam, Sanjit Kumar Pal

**Affiliations:** 1 Wadia Institute of Himalayan Geology, Dehradun, India; 2 Department of Applied Geophysics, Indian Institute of Technology (IIT-ISM), Dhanbad, India

**Keywords:** Geodynamics, Geophysics, Tectonics

## Abstract

The possibility of a major earthquake like 2015 Gorkha–Nepal or even greater is anticipated in the Garhwal–Kumaun region in the Central Seismic Gap of the NW Himalaya. The interseismic strain-rate from GPS derived crustal velocities show multifaceted strain-rate pattern in the region and are classified into four different strain-rate zones. Besides compressional, we identified two NE–SW orienting low strain rate (~ 20 nstrain/a) zones; namely, the Ramganga-Baijro and the Nainital-Almora, where large earthquakes can occur. These zones have surface locking widths of ~ 72 and ~ 75 km respectively from the Frontal to the Outer Lesser Himalaya, where no significant surface rupture and associated large earthquakes were observed for the last 100 years. However, strain reducing extensional deformation zone that appears sandwiched between the low strain-rate zones pose uncertainties on the occurences of large earthquakes in the locked zone. Nevertheless, such zone acts as a conduit to transfer strain from the compressional zone (> 100 nstrain/a) to the deforming frontal active fault systems. We also observed a curvilinear surface strain-rate pattern in the Chamoli cluster and explained how asymmetric crustal accommodation processes at the northwest and the southeast edges of the Almora Klippe, cause clockwise rotational couple on the upper crust moving over the MHT.

## Introduction

The ongoing collision of India and the Eurasian plates since ~ 50 Ma keeps the Himalayan mobile belt seismically active^1–3^. The continued northward movement of the Indian plate against Eurasia resulted substantial stress accumulation across the Himalayan collisional belt which eventually released as small to large earthquakes^4–6^. The great Himalayan arc witnessed many devastating earthquakes in the past like the 1905 Kangra, 1934 Bihar–Nepal, 1950 Assam, 2005 Kashmir, 2015 Nepal and many more indicate that the region is seismically vulnerable^7–10^.

Geodetic studies indicate about half of the movement of the Indian plate (50 mm/a) is getting adjusted across the Himalayan plate boundary faults^11–13^. The Main Himalayan Thrust (MHT) is the detachment fault, which is kinematically locked from the Himalayan Frontal Thrust (HFT) and have a 100 km wide zone of strong interseismic coupling^4,13–15^ towards the Sub and the Lesser Himalaya. Beyond the locking zone and towards the MCT, the elastic strain energy is frequently releasing as small to moderate magnitude earthquakes^4,5,14^. There are studies^16–18^ that suggest the transfer of strain energy from the Higher to the Sub- and to the Frontal Himalaya, where it gets adjusted causing neotectonic deformation of the active fault systems in the frontal Himalaya. Thus the upper crust within the locked zone is accumulating high strain which can exceed its hold-up limit at any time and break its quiescence for a large magnitude earthquake. However, what remains unanswered is the mode of deformation; whether, compressional or extensional or the combination of both is regulating the transfer of strain energy from the Higher to the frontal Himalaya. In fact, there are heightened concerns about a repetition of 2015 Gorkha–Nepal like Major magnitude earthquake in the Northwest Himalaya; particularly, in the Garhwal–Kumaun region. Thus the objective is to study the characteristics of interseismic strain-rate, its pattern, identification of potential strain-rate zones, deformation of local and major thrust systems in aiding or diffusing the strain rates, and finally narrow down to areas where large earthquakes can occur in this Central Seismic Gap zone^19^. Results suggest the existence of multifaceted strain-rate zones, including potential earthquake zones in the Garhwal–Kumaun Himalaya. However, the interplay of strain distribution between these zones also poses uncertainties towards possible occurrences of large magnitude earthquakes in the study region.

### Regional tectonic setup

**Figure 1:**
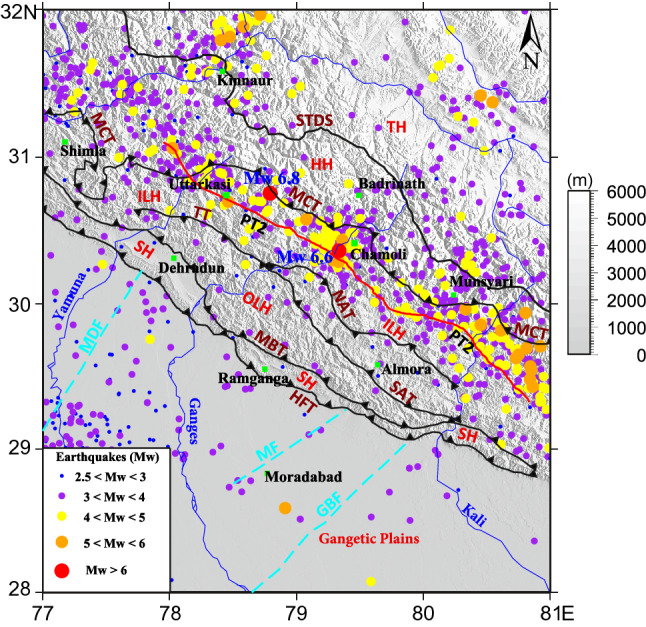
Morphotectonic map of the study area with seismicity. Major plate boundary faults namely Himalayan Frontal Thrust (HFT), Main Boundary Thrust (MBT), Main Central Thrust (MCT), South Tibetan Detachment System (STDS) are marked along with the Mahendragarh–Dehradun Fault (MDF), Moradabad fault (MF), Great Boundary Fault (GBF) and the physiographic transition (PT2). The major morphotectonic zones namely Sub-Himalaya (SH), Outer Lesser Himalaya (OLH), Inner Lesser Himalaya (ILH), Higher Himalaya (HH) and the Tethyan Himalaya (TH) are marked. Colored dots indicate the seismicity of the region with size increasing with magnitude (data from ISC catalog). The basemap is the 3 arc-second elevation from SRTM Digital Elevation Model in greyscale and the map was created by Generic Mapping Tool (GMT) open source software version 5.4.5 (https://www.generic-mapping-tools.org/).

Figure 1 represents the tectonic map and the regional seismicity of the study area. The regional seismicity data is from the International Seismological Centre (ISC) catalog. The earthquake data are converted to moment magnitude (Mw) using the regression relations^20^. Subsurface transverse faults like the Mahendragarh–Dehradun Fault (MDF), Moradabad fault (MF), and the Great Boundary Fault (GBT) extending from the Gangetic plain towards the Himalayan arc are also marked. The Garhwal–Kumaun region in the Northwest Himalaya lies in the central seismic gap between the rupture zones of the Ms 7.8, 1905 Kangra earthquake and the Mw 8.2 1934 Bihar–Nepal earthquake^19,21^. Morphotectonically the study region is divided into four zones; namely, the Sub-Himalayas or the Siwaliks having Quaternary sediment-filled alluvial valleys with topographic height ranges ~ 900–1200 m and is bounded by the Himalayan Frontal Thrust (HFT) at its south and the Main Boundary Thrust (MBT) at its north^22,23^. The low-grade metasedimentary rocks in the Lesser Himalaya along with the Nappe and Klippe structures (Almora Klippe, Nappe and klippes of Lansdowne, Askot etc.) are morphotectonically guarded by the MBT and the Main Central Thrust (MCT) at its south and the northern boundaries respectively^24,25^. In general, the Lesser Himalaya is subdivided into Outer and Inner Lesser Himalaya by the south-dipping Tons Thrust (TT) and the North Almora Thrust (NAT)^22,24–27^. The north-dipping South Almora Thrust (SAT) marks the southern boundary of the Almora Klippe. In this work, we also enquire how the Lesser Himalayan fault system (TT, NAT & SAT) affects the regional upper crustal velocities, strain distribution, and subsequent seismicity. Most of the Himalayan earthquakes are occurring in the Himalayan Seismic Belt (HSB) that extend ~ 100 km from the Physiographic Transition (PT2) towards the MCT in the Higher Himalaya^8,28^. The PT2 is a steep topographic transition that corresponds the downdip edge of the locked portion of the MHT^28,29^. Studies show, majority of earthquakes are occurring at depths ~ 15 km, where the Mid Crustal Ramp (MCR) of the MHT undergo seismic slips^4,8,30^. While at the north of Higher Himalaya the Tethyan Himalayan sequence continues its movement towards the India—Eurasia collisional margin^31^.

## Results and discussions

### Regional crustal velocity and surface convergence

**Figure 2:**
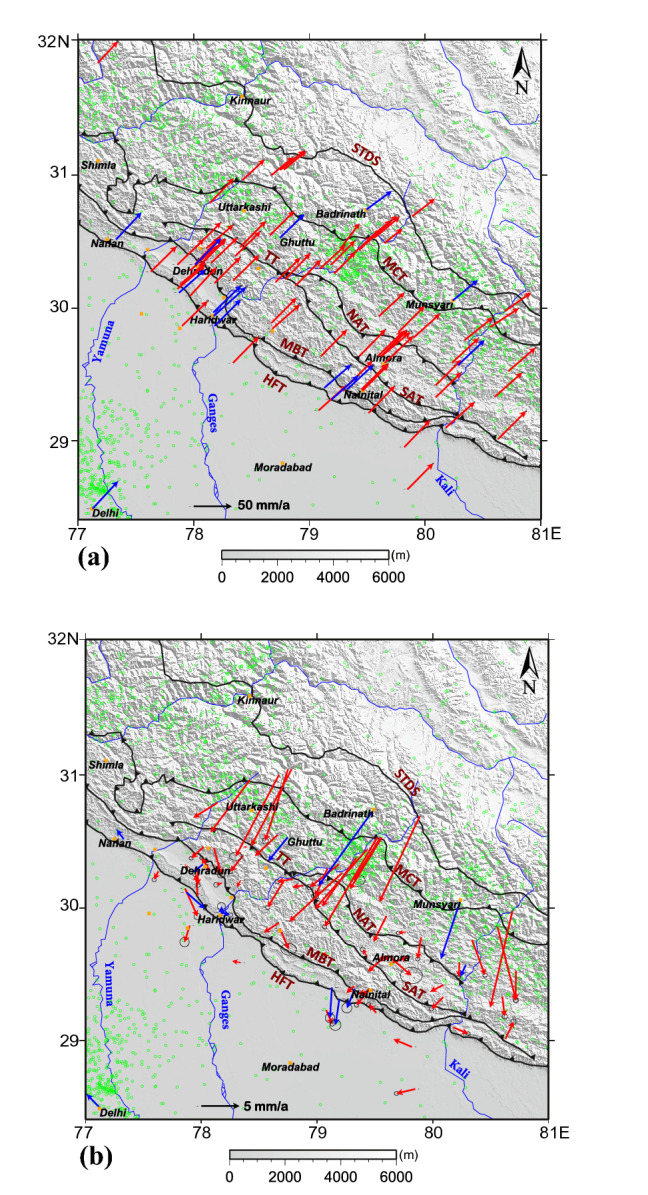
**(a)** Figure shows the velocities of the GPS stations in the Garhwal–Kumaun region in ITRF2008. The blue arrows represent the velocities of WIHG GPS stations whereas the red arrows represent the velocities from published works. Seismicity of the region is marked as green circles. The figure was created by Generic Mapping Tool (GMT) open source software version 5.4.5 (https://www.generic-mapping-tools.org/). (**b**) Figure shows the velocities of the GPS stations in the Garhwal–Kumaun region in the Indian reference frame^32^. The blue arrows represent the velocities of WIHG GPS stations whereas the red arrows represent the velocities from published works. Seismicity of the region is marked as green circles. The figure was created by Generic Mapping Tool (GMT) open source software version 5.4.5 (https://www.generic-mapping-tools.org/).

GPS data from Wadia Institute of Himalayan Geology (WIHG) local network were processed along with the surrounding International GNSS Service (IGS) stations data using the Gamit/Globk software and estimated the site velocities. Details on data and the processing are given in the Data and Methodology section. The estimated regional crustal velocities in the International Terrestrial Reference Frame (ITRF) are shown in Fig. 2a and values are given in Table 1. In the Himalayan mobile belt the crustal velocities are oriented towards the northeast as that of the movement of the Indian Plate^13,14^. However, crustal velocities along the Himalayan arc increases from 48.62 ± 0.10 mm/a in the Garhwal Sub-Himalaya to 49.63 ± 1.05 mm/a in the Kumaun Sub-Himalaya. Whereas, across the arc velocities decreases from 48.81 ± 0.14 mm/a in the south of HFT to 41.24 ± 0.02 mm/a in the Higher Himalaya. While in the Indian reference frame^32^ (Fig. 2b) almost all the stations in the Higher and the Lesser Himalaya situated at the west of river Kali shows relatively larger velocities and oriented towards SW. But stations at the Sub Himalaya (TSW1) and the Frontal Himalaya (HARI) show lower velocities of 1.62 ± 0.10 mm/a and 0.36 ± 0.14 mm/a respectively, which emphasize that the detachment fault MHT is locked towards the Frontal Himalaya. But beyond the locking zone and towards the north, the Lesser and Higher Himalayan crustal velocity scenarios are different. The inner Lesser Himalayan station GHUT (Ghuttu) and the MUNS (Munsyari) station in the Higher Himalayan mobile belt show much higher velocities of 4.02 ± 0.06 mm/a and 9.20 ± 0.02 mm/a respectively. The shortening rate of the Indian crust has been estimated by taking the residual velocity between the Haridwar station situated at the south of HFT and the Hanle station^33^ in the Eurasian plate. Estimated rate shows a 16 ± 0.8 mm/a crustal shortening in the Garhwal Himalaya. The linear surface shortening rates between Higher and the Lesser, Lesser and the Sub and Sub and the Gangetic plains across the Garhwal region are also estimated as 7.38 ± 0.24 mm/a, 2.40 ± 0.02 mm/a and 1.26 ± 0.02 mm/a respectively. The linear surface shortening rate estimated across the Kumaun Himalaya is 17.62 ± 4.6 mm/a. Linear shortening rates of 5.41 ± 0.55 mm/a and 0.72 ± 0.22 mm/a are observed between the Higher and the Lesser and the Lesser and the Gangetic plains across the Kumaun Himalaya respectively (Table 2). Significant surface convergence is occurring between the Lesser and the Higher Himalayas at both Garhwal and the Kumaun regions. But at relatively enhanced rates in the Uttarakashi and the Chamoli regions of Garhwal Himalaya, where elevated seismicity and the occurrences of Moderate Magnitude earthquakes are quite frequent.

**Table 1:** Estimated velocities of GPS stations in ITRF2008 and in the Indian reference frame.

Si. no.	Station name	Longitude	Latitude	ITRF 2008				Indian reference frame		Rho
				East (mm/a)	North (mm/a)	σE (mm/a)	σN (mm/a)	East (mm/a)	North (mm/a)	
1	ALIC	133.88552	− 23.67011	31.39	59.89	0.05	0.06	1.19	35.30	− 0.057
2	BJFS	115.89249	39.60860	30.38	− 10.92	0.04	0.04	− 13.50	− 41.94	0.051
3	JFNG	114.49102	30.51556	33.60	− 10.61	0.13	0.13	− 11.80	− 42.03	− 0.046
4	WUHN	114.35727	30.53165	32.82	− 10.38	0.08	0.07	− 12.53	− 41.82	− 0.079
5	ULAB	107.05233	47.86507	28.60	− 8.25	0.03	0.04	− 8.93	− 41.34	0.024
6	IRKM	104.31625	52.21902	25.04	− 6.59	0.08	0.06	− 9.23	− 40.16	0.042
7	NTUS	103.67996	1.34580	28.69	− 6.98	0.05	0.04	− 15.26	− 40.02	− 0.127
8	LHAZ	91.10403	29.65733	46.03	15.45	0.06	0.06	7.25	− 19.42	0.019
9	URUM	87.60067	43.80795	31.79	5.34	0.05	0.06	0.62	− 29.53	− 0.014
10	NVSK	83.23545	54.84061	25.38	− 0.55	0.03	0.04	2.82	− 35.25	− 0.060
11	LCK2	80.95597	26.91256	37.00	35.33	0.07	0.06	0.07	0.74	− 0.017
12	LCKI	80.95589	26.91244	37.00	35.33	0.07	0.06	0.07	0.74	− 0.017
13	LCK3	80.95564	26.91218	37.00	35.33	0.07	0.06	0.07	0.74	− 0.017
14	HYDE	78.55087	17.41726	40.93	35.05	0.06	0.05	1.00	0.67	0.011
15	IISC	77.57038	13.02117	42.91	34.84	0.03	0.03	1.78	0.57	0.008
16	CHUM	74.75110	42.99850	27.66	2.81	0.03	0.03	1.31	− 31.04	− 0.016
17	POL2	74.69427	42.67977	27.24	4.65	0.02	0.02	0.72	− 29.19	− 0.056
18	DGAR	72.37024	− 7.26968	47.05	33.08	0.04	0.03	2.42	− 0.45	0.016
19	KIT3	66.88545	39.13477	27.33	4.89	0.04	0.04	1.49	− 27.50	0.006
20	REUN	55.57172	− 21.20822	16.79	11.83	0.04	0.03	− 30.87	− 17.42	0.084
21	SEY1	55.47941	− 4.67372	24.87	11.38	0.08	0.07	− 20.28	− 17.85	0.057
22	TEHN	51.33410	35.69728	26.44	20.08	0.02	0.02	3.16	− 7.66	− 0.030
23	BHR4	50.60815	26.20914	30.67	30.29	0.14	0.13	0.85	2.80	− 0.015
24	VOIM	46.79327	− 21.90630	18.58	16.22	0.54	0.46	− 30.67	− 9.78	0.074
25	SOLA	46.40057	24.91068	30.97	29.25	0.03	0.03	1.10	3.42	0.033
26	ISBA	44.43841	33.34142	24.76	27.93	0.03	0.03	1.49	2.93	− 0.043
27	MBAR	30.73788	− 0.60147	25.85	17.62	0.04	0.04	− 18.20	− 0.93	0.062
28	MFKG	25.53997	− 25.80501	17.92	19.65	0.10	0.09	− 34.97	3.87	0.094
29*	MUNS	80.24037	30.06049	32.09	25.91	0.02	0.02	− 3.26	− 8.61	− 0.010
30*	HARI	78.18085	29.86910	35.23	33.71	0.11	0.10	− 0.30	0.20	− 0.006
31*	TSW1	78.01389	30.32883	34.20	34.11	0.02	0.02	− 1.26	− 1.03	− 0.012
32*	BIHA	77.87416	30.11874	36.21	32.88	0.11	0.10	1.24	− 6.01	− 0.065
33*	SHAM	77.32838	30.51860	33.38	34.46	0.02	0.02	− 0.92	1.27	− 0.007
34*	GHUT	78.74743	30.53100	32.31	31.44	0.03	0.04	− 2.55	− 3.11	− 0.008
35*	DELH	77.12626	28.48239	33.40	36.05	0.21	0.18	− 1.77	1.85	− 0.028
36*	BDRI	79.49346	30.74292	33.38	24.36	0.09	0.07	− 2.42	− 10.09	0.010
37*	PTH2	80.28556	29.57304	34.87	33.01	0.13	0.12	− 0.71	− 1.52	0.006
38*	CHDI	78.17472	29.94351	42.65	38.35	1.49	1.38	− 0.02	1.25	0.001
39*	MNSI	78.16934	29.96968	36.57	35.30	0.99	0.78	1.20	− 0.36	0.001
40*	SYAT	79.32601	29.38969	34.35	31.95	1.88	1.84	− 1.03	− 2.49	0.016
41*	PTPN	79.19309	29.31340	34.87	31.02	2.06	2.00	− 0.51	− 3.41	0.010
42*	RMGR	79.12907	29.40093	35.07	30.41	1.76	1.76	− 0.25	− 4.01	0.006

*Mark indicates the WIHG GPS stations.

**Table 2:** Estimated linear shortening rates across the Garhwal and the Kumaun Himalaya.

	Linear shortening rates (mm/a)	
	Garhwal Himalaya	Kumaun Himalaya
Overall	16.01 ± 0.80	17.62 ± 4.60
Higher—Lesser Himalaya	7.38 ± 0.24	5.41 ± 0.55
Lesser—Sub Himalaya	2.40 ± 0.02	0.72 ± 0.22
Sub Himalaya -Gangetic plains	1.26 ± 0.02	
Across TT	1.30 ± 1.22	
Across NAT		0.68 ± 0.65
Across SAT		1.44 ± 0.48
Across Almora Klippe (SE)		2.12 ± 0.55
Across Almora Klippe (NW)		2.64 ± 1.66

Apart from main thrusts, there are local thrusts; such as Tons Thrust, North Almora Thrust, and the South Almora Thrust. These thrusts are actively regulating the surface strain pattern in the Garhwal–Kumaun Himalaya. The south-dipping Tons thrust separates the inner and the outer Lesser Himalaya along with the North Almora and the South Almora thrusts. And also, the north and the south Almora thrusts demarcate the northern and the southern boundaries of the Almora Klippe^26,27^ (supplementary Fig. 1). The Almora Klippe on which the Higher Himalayan crystalline thrusts overlayed on the Lesser Himalayan metamorphic rocks created complex strain patterns^24,25^. The south-dipping NAT and the north-dipping SAT between the MBT and the MCT oppose the northward movement and make complex deformation of the Kilppe. Low seismicity is observed across the Almora klippe which indicates that the region is locked and the local thrust system also adjusts accordingly.

### Surface strain-rate analysis and the classification of different strain-rate zones

**Figure 3:**
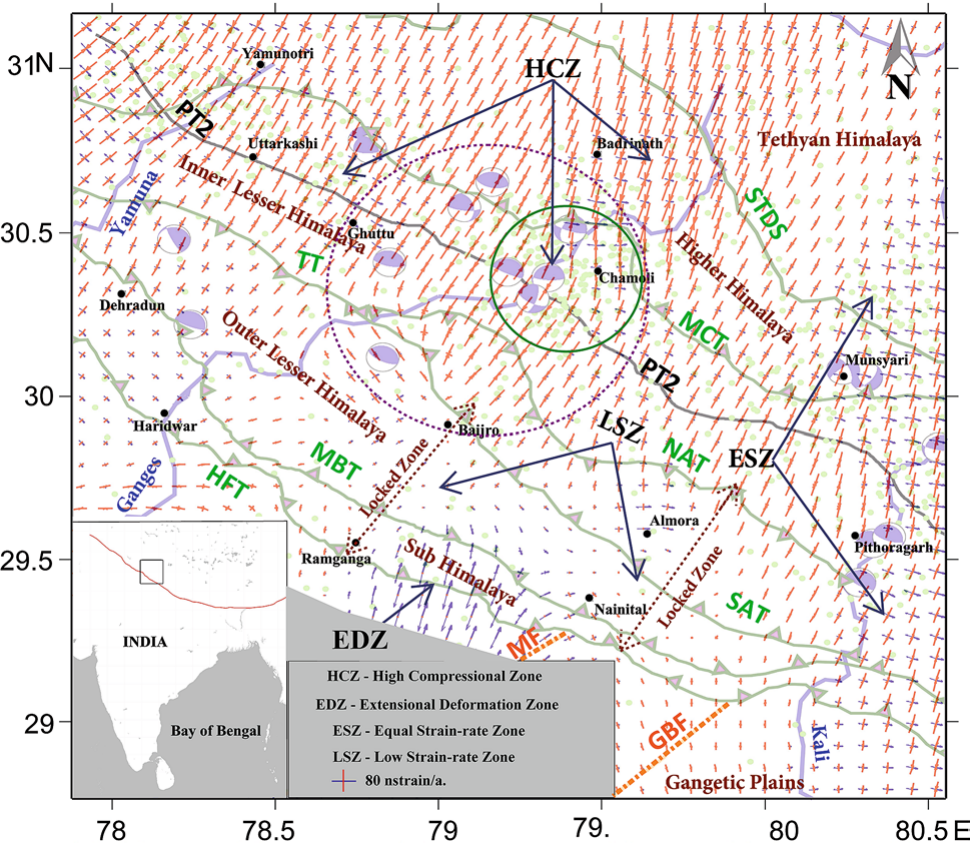
Overall strain rates in the Garhwal–Kumaun region. The red arrows in the strain vectors indicate the compressional axis and the blue arrow shows the extensional component. High compression zone (HCZ), Extensional deformational zone (EDZ), Equal strain-rate zone (ESZ), Low strain-rate zone (LSZ), and the locked zone are marked. The Chamoli earthquake cluster is marked by the green circle. Seismicity of the region is given in green dots and available focal mechanism solutions (Supplementary Table. 1) are also marked as beach balls. The wider violet dotted circle represents the curvilinear deformation field focusing to the Chamoli cluster. The black rectangle given in the inset map at the left bottom corner of the plot indicates the study area. Base map of the plot was created using Generic Mapping Tool (GMT) open source software version 5.4.5 (https://www.generic-mapping-tools.org/) and the further modifications are done using the Global Mapper software (https://www.bluemarblegeo.com/global-mapper/).

The existence of varied surface accommodation rates in the study area poised us to look into the regional strain-rate pattern, seismicity, and the available fault plane solutions. Crustal strain rates in the study region were calculated from the GPS estimated crustal velocities based on the modified least square approach as explained by Teza et al.^34,35^. Details of the strain-rate calculations are given in the Data and Methodology section. Analysis shows the existence of multifaceted strain-rate zones, where local and regional thrust systems have pivotal roles in regulating the overall regional seismicity distribution. Figure 3 explains the strain- rate vectors and we identified four types of surface deformation zones in the study area, namely (1) the High compressional zone (HCZ) -in the active seismic belt close to the MCT. (2) the Extensional deformation zone (EDZ) -mainly located in the Frontal Himalaya adjoining to the transverse Moradabad Fault (MF) (3) the Equal strain-rate zone (ESZ), having nearly equal compressional and extensional components -mainly seen at the east of the river Kali and (4) the Low strain-rate zones (LSZ) -observed as isolated corridors in the Ramganga-Baijro and the Nainital-Almora region.

In the HCZ, higher strain rates are observed from the north of TT and the NAT. The HCZ in the Garhwal—Kumaun Himalaya witnessed Light to Strong Magnitude earthquakes (supplementary Fig. 2a) and are mainly dominated by thrust fault mechanisms with their fault plane dipping towards NNE-SSW^36,37^. The presence of steeply dipping (> 16°)^30,38^ MCR plausibly enhances the slip of overburden over the MHT and thereby release the lithostatic stress as frequent earthquakes. The observed surface strain-rate vectors increases from the north of Ton thrust in the inner Lesser Himalaya, where the underlying MHT dips steeply right below the physiographic transition (PT2). Moreover, we had seen relatively larger crustal accommodation rate across the Kumaun Himalaya compared to the Garhwal Himalaya. Varied crustal accommodation rates are proxies that represent variable dip slips along the MHT which is reflected in the observed asymmetric surface strain vectors. Relatively smaller crustal accommodation is taking place in the HCZ of the Garhwal Himalaya through intermittent small to strong magnitude earthquakes owing to the relatively small seismic slips over a gentle MCR. Hence the accumulated elastic strain energy would be insufficient for a relatively larger earthquake between its Lesser and the Higher Himalaya. However, in the Kumaun region the seismicity rate is relatively less compared to the Garhwal region. Particularly at its inner Lesser Himalaya, where the reduced brittility of the rocks from brittle to semi-brittle enhances greater crustal accommodation which is adjusted as aseismic slips over a relatively steeply dipping MCR.

In general, at the south of HCZ the PT2 demarcates the southern boundary of earthquakes and the temperature at the subsurface of MHT (~ 300–350 °C) corresponds to the PT2 also favors the transition of rocks from brittle to semi-brittle^28^. Besides, there is also a reduction in effective rock stress aided with the increase of subsurface pore fluid pressure^28,29^. These processes enhance earthquake activities in this HCZ and hence validate higher crustal strain-rate vectors. We also observed the Chamoli earthquake cluster in the HCZ (encircled by green colour) with a maximum strain-rate of ~ 150 nstrain/a, and the same will be discussed separately in the subsequent section. Beyond the Chamoli cluster, high compressional strain is continuing towards Badrinath in the NNE direction with a maximum strain-rate of ~ 200 nstrain/a. High compressional strain (~ 150 nstrain/a) is visible throughout the MCT zone and shows good agreement with the seismic activities.

From Fig. 3 it is evident that the direction of compressional strain is in general towards NNE, which is same as that of the present-day movement of the Indian Plate. But it is interesting to note the existence of an extensional deformation zone (EDZ, ~ 100 nstrain/a) in the frontal part of the Kumaun Himalaya where many active faults like Dhikala, Pawalgarh^39^ are present along with the NE–SW oriented transverse Moradabad fault. Trench excavation studies suggest that these frontal active fault systems are responding to the strain transfer process through long-term aseismic deformation^40,41^. The southward orientation of extensional strain vectors from the Higher Himalaya towards the frontal part also elucidates that the strain energy is getting transferred towards the frontal active fault systems and subject the faults to aseismic deformation and their southward propagation. In this process the detachment fault MHT acts as a conduit for the strain energy transfer to the frontal Himalaya. However, there is a possibility that the transferred elastic energy might be acting beyond the HFT towards the south and contributing to the local seismicity around the National Capital Region (NCR) of Delhi.

In the Equal Strain-rate zone (ESZ, ~ 100 nstrain/a), the compressional and extensional strain-rates are acting on the rock mass more or less equally. This zone is mainly seen throughout the eastern part of the Kali river near Nepal Himalaya. In general, the extension or along the arc movements are mainly seen in the frontal part of the Garhwal–Kumaun Himalaya. However, in the ESZ case, the extensional phase is present not only all along the HFT but also towards STDS with an equal amount of compressional strain component. This shows strain partitioning is predominant in this region and can be linked with larger across the arc convergence rate as well as along the arc oblique movement at the east of river Kali and adjacent to the Nepal Himalaya.

**Figure 4:**
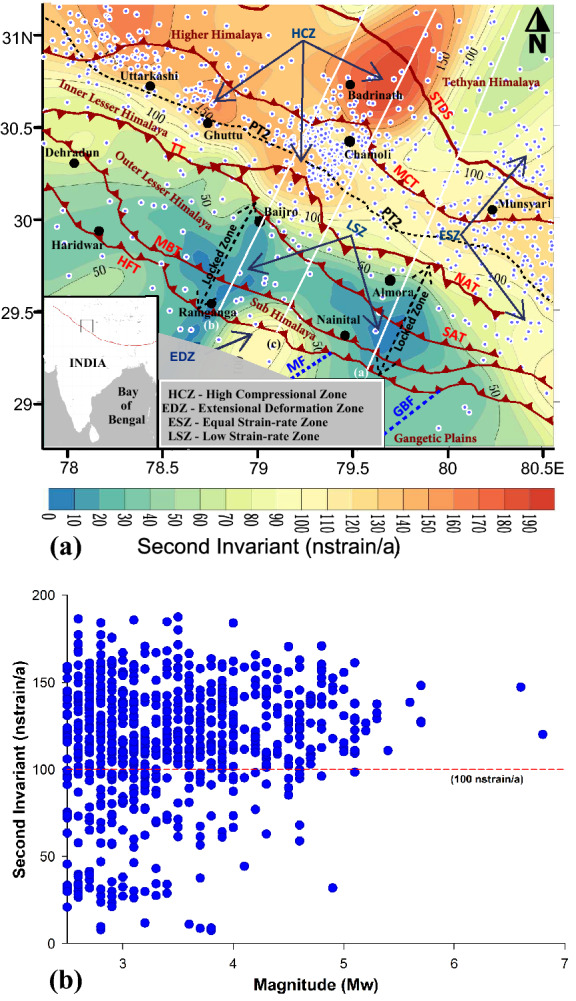
**(a)** Second invariant strain map of the Garhwal–Kumaun Himalaya. Coloured contours represent the total strain rates in the region as a scalar quantity. High compression zone (HCZ), Extensional deformational zone (EDZ), Equal strain-rate zone (ESZ), Low strain-rate zone (LSZ), and the locked zone are marked. Three profiles are plotted in the figure and their explanations are given in Fig. 5. The black rectangle given in the inset map at the left bottom corner of the plot indicates the study area. The base map was created using the Surfer software (https://www.goldensoftware.com/products/surfer) and further modifications are done in Grapher software (https://www.goldensoftware.com/products/grapher). (**b**) Second invariant of strain-rate above and below the 100 nstrain/a barrier is plotted against the earthquake size.

Interestingly, there are two corridors of low strain-rate zones (LSZ) visible in the vector strain map (Fig. 3). The LSZ (< 20 nstrain/a) is also clearly observable from the second invariant of strain as shown in Fig. 4a. These corridors include one near the Ramganga reservoir in the Sub-Himalaya and extends up to Baijro in the outer Lesser Himalaya and the other is at the Nainital-Almora Region. From Figs. 3 and 4a it is evident that the Tons thrust and the north Almora thrusts which separate the outer and the inner Lesser Himalaya act as a structural barrier between the high compressional zone and the extreme low strain-rate zones. Here the strain-rate increases from ~ 100 nstrain/a to a maximum value of ~ 200 nstrain/a towards Badrinath region at the north of MCT. Feeble strain rates in the Sub and the outer Lesser Himalaya are plausible because of two reasons. (1)There may be no net force acting on the frontal fault systems of the region or (2) High frictional force is acting on the locked detachment fault, which might be opposing the surface deformation. The gently dipping MHT and the Moho with greater frictional force in the Sub and the outer Lesser Himalaya obstruct seismic activity and hence the zone experiences lesser seismic deformation (supplementary Figs. 1b and 2b). In the Ramganga—Baijro window, the nature of strain starts changing from Baijro in the proximity of NAT towards further north, and ultimately it converges towards the Chamoli region. Whereas in the Nainital—Almora corridor, the low strain zone extends up to the south of NAT.

The intercomparison of geodetic strain-rate with seismicity as reported^42^ earlier suggests that low strain rate zones present within the locking width are more likely to produce a great earthquake. Figure 4b shows the interseismic geodetic strain-rate plotted against the earthquake size in our study area. Most of the earthquakes in the study region are occurred in the high strain rate zones (> 100 nstrain/a) including the two significant earthquakes, the 1999 Mw 6.6 Chamoli and the 1991 Mw 6.8 Uttarkashi earthquakes^8^. Figure 4b thus supports that the high compressional zone which lies in the Himalayan seismic belt close to the MCT, shows Moderate to Strong Magnitude earthquakes having recurrence intervals of ~ 5 to 7 years (supplementary Fig. 2a). Thus the region away from the locked zone and towards the Himlayan Seismic Belt (HSB) the stored elastic strain energy is sequentially draining and thereby weakening the occurence of large earthquakes as long as the region maintains its current level of seismicity.

The nearly NE–SW oriented low strain-rate corridors observed in the Lesser and the Sub-Himalaya (Fig. 4a) are lying over the gently dipping MHT and the subsurface duplex structures within the locked portion. This region lies south of the PT2 is considered highly locked and it shows strong interseismic coupling^14,28^. Figure 4b shows that, the region with low strain rates are not witnessed any significant events (Mw > 5) in the last 50–60 years (Also see supplimentary Fig. 2b). Paleo seismological studies also suggest that there are no transverse rupture zones of any great historical earthquakes^43^ in the outer Lesser and the Sub-Himalayan sections within the LSZ. Had it been, then there would have dissipation of strain energy as seismic or aseismic slips. Besides, the low-grade metasedimentary rocks present in the Outer Lesser Himalaya are relatively brittle and could accommodate little elastic strain energy. But the heightened concern is about the possibility of a Major or a Great earthquake as there are evidences of many such earthquakes reported from the frontal part within the locked portion of the great Himalayan arc^4,40,41^. The estimated recurrence interval of such a Mw 7.0 event in the Himalayan region is about 100 years^4^. Thus when the region exceeds its elastic limit then a large earthquake can produce a large rupture extending towards the HFT and the Gangetic plain, where secondary seismic effects like liquefaction^10^ or cavity formation can cause great havoc in the densely populated foothills. Thus the regions marked as interseismic low strain rate corridors in Figs. 3 and 4a have the potential for a large earthquake. The estimated surface locking width of Ramganga-Baijro and Nainital-Almora LSZ is ~72 and ~75 km respectively from the HFT. However considering the possible recurrence of 1905 Kangra earthquake, then the Sub- and the Frontal Himalaya are on long over due for such an earthquake. This implies there are strain diffusing factors that could put off or create uncertainties on the occurrences of such events in these regions due to the existence of EDZ.

The second invariant of strain (Fig. 4a) as calculated from the principal strain components also identify the other three strain-rate zones apart from the LSZ. These estimated strain-rates are well-matched with the strain model of Kreemer et al. 2014^44^. The second invariant clearly shows the nature of the extensional deformational zone (EDZ) which appears sandwiched between the earlier mentioned NE–SW orienting low strain-rate corridors (< 20 nstrain/a) and predominantly seen in the Sub and the Frontal Himalaya. The change in the surface area also supports extensional zone having positive eigenvalue contours (6 × 10^–08^/a) near the MF in the EDZ, while compressional zones have negative eigenvalue contours near the MCT (Supply. Figure 3). The EDZ is the strain energy diffusion zone, where extensional deformation happens through the transfer of strain energy from the high strain-rate region in the Lesser Himalaya and extends towards the frontal Himalaya. Greater dissipation of strain energy takes place at the frontal part where the active fault systems undergo along the arc aseismic deformation. Interestingly, transverse fault like the Moradabad fault (MF) that is connected with the frontal EDZ plausibly getting a share of the elastic strain energy at the rate of ~ 100 nstrain/a; which in turn get released as Minor to Moderate magnitude earthquakes in the NCR of Delhi. Gaur (1993)^45^ explained how such frontal transverse faults segement the overthrusting Himalayan arc and facilitate strain relaxation through staggered slips over a period of time. Thus the  significance of sub and frontal EDZ in deferring the much anticipated large earthquakes in the locked portion of the region cannot be ruled out.

### Correlation between strain-rate and the topography

The correlation of strain-rate profile with topography would explain the mechanism of strain-rate transfer from the Higher to the Frontal Himalaya. As we traverse north across the thrust systems in the Garhwal–Kumaun Himalaya the topography increases, hence the gravitational potential energy^29^. Thus a positive correlation between the topography and the strain-rate is expected. Similarly, the subsurface duplex systems in the Lesser Himalaya are also influencing the seismicity and the deformation characteristics in the region^28^. Thus we attempt to understand the mechanism of strain transfer towards the frontal, by unraveling (1) how the strain-rate changes as one moves across-the-thrust systems? (2) the role of subsurface Lesser Himalayan duplex system on across-the-thrust strain-rate and (3) how the overburden topography affects strain-rate distribution in terms of Gravitational potential energy change (GPE)?

**Figure 5:**
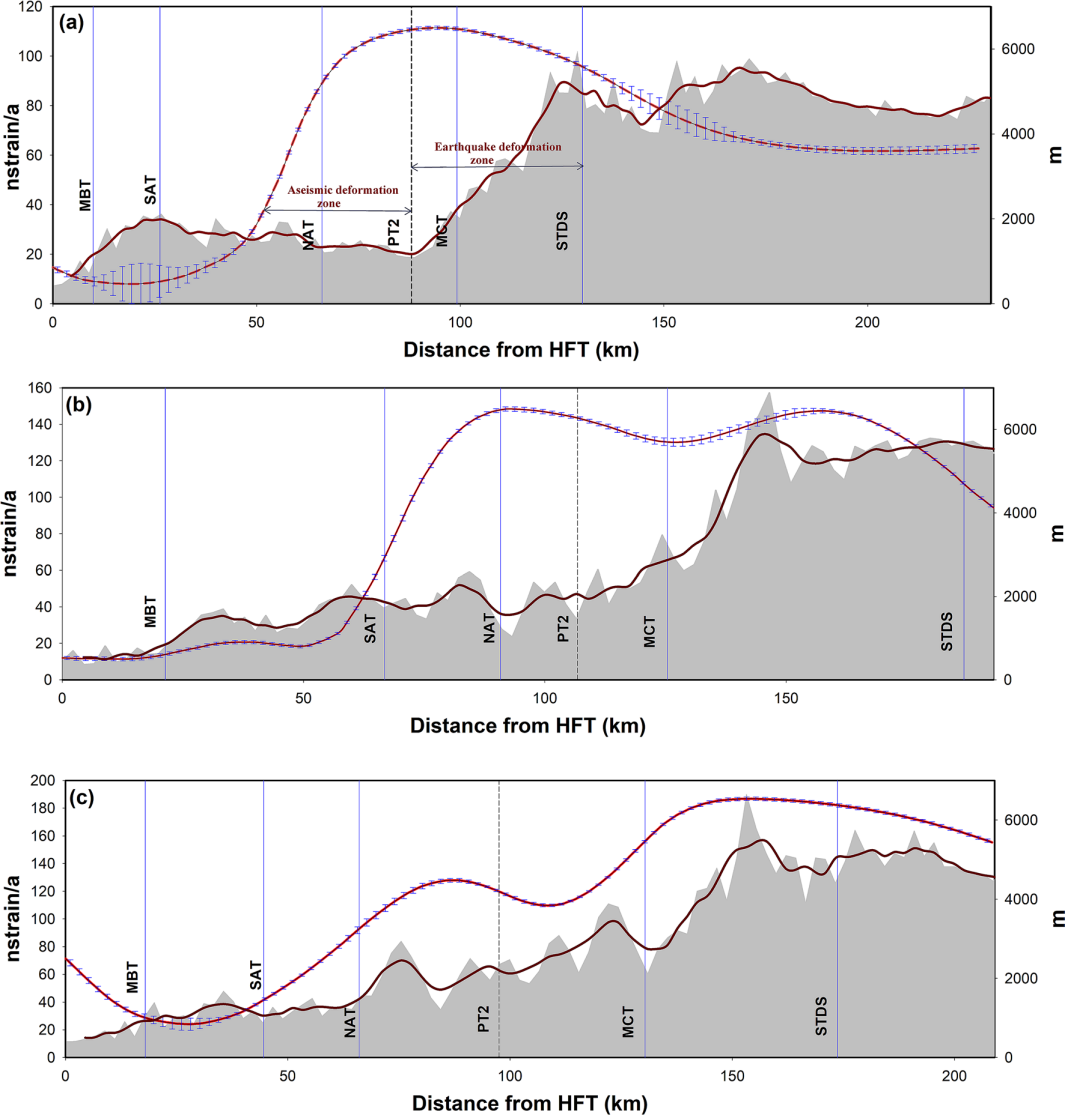
Strain-rate Vs Topography across three profiles. (**a**) across Nainital-Almora in LSZ, (**b**) Ramganaga-Baijro in LSZ and (**c**) Ramnagar-Badrinath begins from EDZ. The red lines with error bars indicate the strain rates and their uncertainties along the profiles. The grey areas represent the elevations and brown lines show the mean elevations along the profiles.

Three strain-rate profiles are taken across the thrust systems in the LSZ and the EDZ as shown in Fig. 4a. Figure 5a,b represent the strain-rate profiles across the Nainital—Almora and the Ramganga—Baijro LSZ corridors respectively; and Fig. 5c shows the strain-rate across the Ramnagar-Badrinath profile. We observe a mismatch between the strain-rate and the topography in both profiles (a) and (b); particularly, in the Lesser Himalaya where the strain-rate changes from low to high values and seen out-of-phase with the topography. However, in the Higher Himalaya reasonable correlation exist between the strain-rate and the first order topography and seismicity. While moving towards the mountain front along the profile (a) the topography falls from PT2 at the south of MCT; but the strain-rate still stands high at PT2 and it falls only at NAT. Whereas, for the profile (b) the high strain-rate further continuing up to the SAT. As we observed that the topography is not well correlated with the strain-rate changes in the Lesser Himalayan part of the profiles (a) and (b). Hence the GPE aided strain transfer from the Higher to the frontal Himalaya is not prominent in these sections. Thus instead of a single mid-crustal sub-surface ramp, an alternative model involving Lesser Himalayan sub-surface duplex system has been considered to explain the strain transfer processes. So that, the duplex systems are significant in keeping the strain rates higher in the inner Lesser Himalaya even in the absence of seismicity, but efficient to hold and transfer the strain. Thus from Fig. 5a,b it is evident that the interseismic strain adjustments are mostly occurring in the Lesser Himalayan region and marked as aseismic deformation zones in the profiles and the same is observed at the south of PT2, where the strain energy transfer is happening at different time scales. It is also observable from Fig. 4a that 100 nstrain/a is the threshold, from where the strain-rate trend falls towards the frontal part. The TT and the NAT situated at this strain- rate threshold act as structural barriers. The NAT regulates the transfer of strain energy from the HCZ towards the Lesser and the Frontal Himalaya along the low strain-rate corridors. Thus the local thrust systems like the TT, NAT, and the SAT and their aseismic deformation modulate the overall strain partitioning in the region.

It is also inferred that, if there were no local thrust systems like the NAT or the SAT and the connected duplex systems, then the strain-rate could have been correlatable with the mean topography. This is observed for the case of profile (c) that extends from the EDZ in the frontal part to the HCZ towards the north. We can observe a relatively better correlation between the mean topography and the strain-rate in the Ramnagar-Badrinath profile. Here gradual decrease in the strain rates from the Higher Himalaya towards the extensional frontal Himalaya can be observed, therefore indicating relatively lesser influences of NAT and the Lesser Himalayan duplex system compared to the profiles (a) and (b) in the low strain-rate corridors. Mean topography and the long-wavelength GPE are highly correlatable here and the GPE aided strain transfer towards the frontal EDZ is the dominant mechanism of strain energy transfer in this section.

### Curvilinear strain-rate pattern and the Chamoli earthquake cluster

Chamoli region in the Garhwal Higher Himalaya is situated in the HCZ (encircled by green colour in Fig. 3) and has been known for its high seismicity rate. More than 500 shallow earthquakes are recorded above the MHT depth in this region after the significant Mw 6.6, 1999 Chamoli earthquake^46^. Clustering of seismic events (green dots, in Fig. 3) along with high NE compressional strain with a minor perpendicular extensional component are observed in this region. Caldwell et al. 2013, Mahesh et al. 2012, Rawat et al. 2014, Kanna and Gupta 2020^30,46–48^, suggest the presence of free fluids along the interface of the mid crustal ramp (MCR) beneath the Chamoli region. A low seismic velocity layer was identified and inferred as sub-surface fluid layer having a low frictional coefficient (µ = 0.6–0.7)^30,47,49^ contributes to the enhanced seismicity. Fault plane solutions show strike-slip earthquakes close to the north of the MCT zone; however, thrust solutions are also seen between PT2 and the MCT zone. Although, the presence of sub-surface fluid layer is a sufficient reason to explain the frequent occurrences of small or even minor earthquakes, but not enough for the generation of a great or even significant magnitude earthquake like the Chamoli earthquake of 1999. Thus it is imperative to know how the strain adjustment of local and other thrust systems around the Chamoli cluster could contribute to larger earthquakes and clustered seismicity.

Strain-rate analysis in and around the Chamoli region shows an interesting curvilinear pattern (Encircled by violet dots in Fig. 3). Here the deformation field starts from Baijro in the Inner Lesser Himalaya at the western edge of Almora Klippe, where the TT terminates. The NE trending compressional strain vectors surpass the NAT and converge towards the Chamoli earthquake cluster. As mentioned earlier, in terms of crustal scale, the overall accommodation rate between the Lesser and the Higher Himalayas is asymmetric in the Garhwal and the Kumaun region, which is 7.38 ± 0.24 mm/a and 5.41 ± 0.55 mm/a respectively.

**Figure 6:**
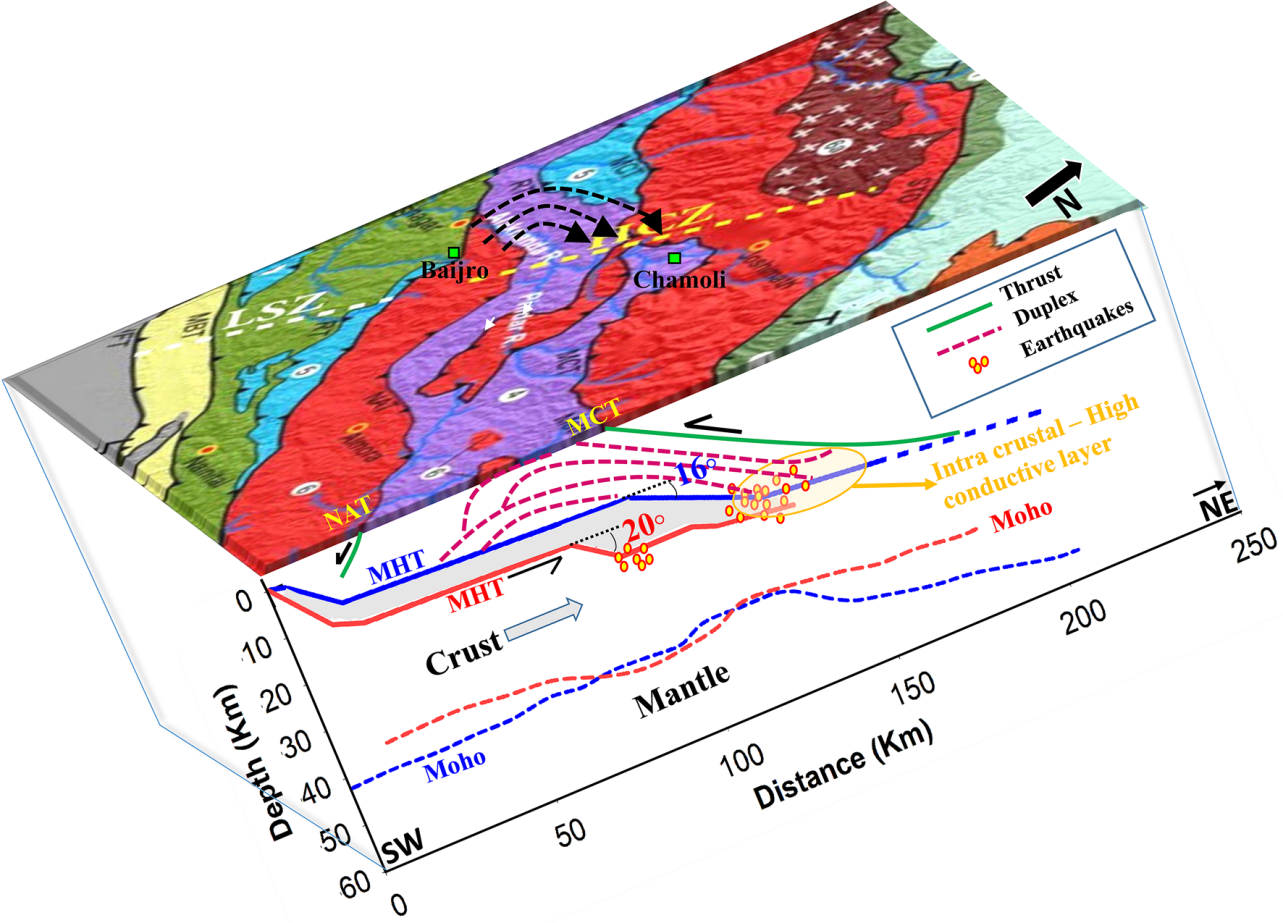
Schematic diagram shows the upper crustal rotation of the Almora Klippe. The look direction of the diagram is from east to west. The overlyed geological map is taken from Thakur et al., 2018^27^. Blue bold line indicate the MHT across northwestern part of the Almora Klippe^30^ and the Red bold line indicates the geometry of the MHT in the southeastern part of the Almora Klippe^50^. Moho geometry of both profiles are indicated in blue and red dash lines. The intra-crustal high conductive layer^47^, Lesser Himalayan duplex system, Main thrusts, and the earthquakes are also plotted in the depth section. The plot was created using the Sigma plot software version 14.5 (http://www.sigmaplot.co.uk/products/sigmaplot/) and further modification are done in microsoft office.

Apart from the crustal-scale accommodation process of MHT, the contribution of local thrust systems toward modulating the Chamoli strain pattern also has to be considered. The linear crustal accommodation rate across the TT in the Garhwal Himalaya is 1.30 ± 1.22 mm/a (Table 2), while the same is 2.12 ± 0.55 mm/a across the Almora Klippe at the eastern Kumaun region. Similarly, across the NAT and the SAT the accommodation rates are 0.68 ± 0.65 mm/a and 1.44 ± 0.48 mm/a respectively. However, larger linear crustal accommodation rate (2.64 ± 1.66 mm/a) is taking place at the western edge of the Almora Klippe between Baijro and Chamoli. Extended level of seismicity from MCT towards Baijro and greater deformation in this region suggest that the local thrusts (NAT and SAT) are asymptotically connected to the gently dipping MHT as compared to the eastern part of the Almora Klippe^30,50^ (Fig. 6). This observed asymmetry in the crustal accommodation processes over a gently and steeply dipping ramps at the northwest and the southeast edges of the Almora Klippe, respectively; creates a clockwise (NE orienting) rotational couple and variable slips of the upper crust over the MHT (Fig. 6). This rotational couple creates a curvilinear surface strain pattern with their point of convergence is directed towards the Chamoli region. The subsequent release of strain energy owing to the rotational block movement could be the plausible reason for a Mw 6.6 Chamoli type earthquake and the sub-surface fluidity  might have aided the rotational slip and the consequent clustered seismicity.

## Conclusion

Studies on the inter-seismic crustal deformation and strain-rate pattern in the Garhwal–Kumaun Himalaya, in the context of an anticipated 2015 Gorkha–Nepal like Major magnitude earthquake, reveal four types of strain-rate zones; namely, (1) High compressional zone (HCZ), (2) Extensional deformation zone (EDZ), (3) Equal strain-rate zone (ESZ) and (4) Low strain-rate zones (LSZ). The HCZ (~ 150 nstrain/a) corresponds to the zone of active seismicity close to the MCT. A curvilinear strain-rate pattern is observed in the Chamoli cluster which is attributed to the asymmetry in the crustal accommodation processes over a gently and steeply dipping ramps at the northwest and the southeast edges of the Almora Klippe, respectively. This has created a clockwise rotational couple on the upper crust moving over the MHT. The variable slips of the NAT and the SAT regulate the shallow crustal accommodation processes and sustained the rotational slip aided by sub-surface fluids caused the Chamoli earthquake cluster. We identified two NE–SW corridors of LSZ (~ 20 nstrain/a); namely, Ramganga-Baijro and the Nainital-Almora in the Lesser and the Sub-Himalaya, where the surface locking width is ~72 and ~75 km respectively. This highly locked LSZ does not show any surface rupture and had not witnessed any great earthquakes, but the upper crust is strained and has the potential for large earthquakes. However, strain reducing EDZ is seen sandwiched between the LSZ; particularly, in the frontal Himalaya where the GPE aided transferred strain from the HCZ zones gets diffused by the deformation of frontal active fault systems and transverse structures like the Moradabad fault. This causes more ambiguity towards the possible occurrence of large Sub- and Frontal earthquakes in the Garhwal–Kumaun region. The TT and the NAT thrust systems, stand at the threshold of 100nstrain/a, act as structural barriers in the strain transfer processes from the MCT zone to the southern thrust systems.

### Data and methodology

GPS data from 42 stations (details are in Table 1) including data from WIHG local network and IGS (International GNSS Service) for a span of eight years from 2010 to 2017 were used in this study. The data were processed using the GAMIT/GLOBK software version 10.6^51,52^ in the ITRF 2008 reference frame and the position coordinates were estimated by stabilizing surrounding IGS stations. Ocean loading effects of the sites in the GPS data were corrected using the ocean tide model FES2004 and the corrections in the displacements associated with the earth's solid tides were also removed by applying the tidal model IERS03. Atmospheric loading effects and the tropospheric corrections at sites were removed using the atmospheric loading data and the global mapping function model respectively. Generated long time series of each station coordinates are corrected for outliers and velocities were estimated in the International Terrestrial Reference Frame (ITRF) (Table 1). We also used the data from published works^14,33,53–56^ along with our data. All the published data are converted to the ITRF 2008 reference frame and estimated the velocities in the Indian reference frame using the pole of rotation of Ader^32^. The linear surface shortening rates across each fault are calculated from the horizontal velocity residuals. Residual velocities of stations situating north and south of each fault zone are calculated from the Indian reference frame velocities^32^.

Crustal strain rates in the Garhwal–Kumaun region were calculated from the GPS estimated crustal velocities based on the modified least square approach explained by Teza et al.^34,35^. We have excluded the stations having velocity uncertainties of more than 2σ from the surface strain estimation. We choose a local grid of spacing 7.5 km and defined the scale factor (15 km). Local strain rates at each grid node are estimated using the modified least square approach and a weight function is used for the error reduction. The principal strain components are estimated and the resulting eigenvalues represent the strain values at each grid node. The second invariant of horizontal strain rate is calculated using the equation

$${\text{SR}} = \sqrt {e1^{2} + e2^{2} }$$

where e1 and e2 are the principle strain components. The second invariant of the strain rate represents the total strain as a scalar quantity^44,57^.

The seismicity data of the study region were collected from the ISC catalog and converted to the moment magnitude (Mw) using the regression relations^20^. The earthquake data of the study region were classified into two classes; earthquakes above and below 100 nstrain/a zones. The earthquakes in the both classes are subdivided according to their size (Mw) and the recurrence interval for each class of earthquakes are calculated (Supply. Figure 2a,b). The earthquake and the strain invariant data sets of the study region are brought in to a common grid frame. Then for each grid, we identified the different class of earthquakes present and its corresponding second invariant of strain rates (Fig. 4b).

## Supplementary Information

Supplementary Information.
