# Author Correction: Crustal velocity and interseismic strain-rate on possible zones for large earthquakes in the Garhwal–Kumaun Himalaya

**DOI:** 10.1038/s41598-021-02190-6

**Published:** 2021-11-15

**Authors:** John P. Pappachen, Rajesh Sathiyaseelan, Param K. Gautam, Sanjit Kumar Pal

**Affiliations:** 1 Wadia Institute of Himalayan Geology, Dehradun, India; 2 Department of Applied Geophysics, Indian Institute of Technology (IIT-ISM), Dhanbad, India

Correction to: *Scientific Reports* https://doi.org/10.1038/s41598-021-00484-3, published online 28 October 2021

The original version of this Article contained an error in the Acknowledgments section.

"The authors would like to thank the Director, Wadia Institute of Himalayan Geology, Dehradun for providing the necessary facilities and encouragement to carry out this work. Author J.P.P is thankful for the research fellowship from WIHG-ISRO/IIRS project. Comments and suggestions from anonymous reviewers have helped to improve the manuscript. We are thankful to the International GNSS Service (IGS) for the GPS data. We also acknowledge International Seismological Centre (ISC) and Global CMT for the seismicity and focal mechanism data. SRTM data^58^ is used for generating topographic plots using Generic Mapping Tool (GMT)^59^. The manuscript is WIHG Contribution No. WIHG/2021/02/08."

now reads:

"The authors would like to thank the Director, Wadia Institute of Himalayan Geology, Dehradun for providing the necessary facilities and encouragement to carry out this work. Author J.P.P is thankful for the research fellowship from WIHG-ISRO/IIRS project. Comments and suggestions from anonymous reviewers have helped to improve the manuscript. We are thankful to the International GNSS Service (IGS) for the GPS data. We also acknowledge International Seismological Centre (ISC) and Global CMT for the seismicity and focal mechanism data. SRTM data^58^ is used for generating topographic plots using Generic Mapping Tool (GMT)^59^. The manuscript is WIHG Contribution No. WIHG/0137."

The original Article has been corrected.

