# Supplementary material for: Crustal velocity and interseismic strain-rate on possible zones for large earthquakes in the Garhwal–Kumaun Himalaya
Source: Sci Rep. 2021 Oct 28;11:21283. doi: 10.1038/s41598-021-00484-3 (PMC8553753; doi:10.1038/s41598-021-00484-3)
Supplement: Supplementary file 1 — Supplementary Information. [file 41598_2021_484_MOESM1_ESM.pdf]

Crustal velocity and interseismic strain-rate on possible zones for large earthquakes in the Garhwal-Kumaun Himalaya

John P Pappachen[1,2], Rajesh Sathiyaseelan[1*], Param K Gautam[1] and Sanjit Kumar Pal[2]

[1]Wadia Institute of Himalayan Geology, Dehradun

[2]Dept. of Applied Geophysics, Indian Institute of Technology (IIT-ISM), Dhanbad

[*]Corresponding author: satraj@wihg.res.in

Supplementary Figures and Tables

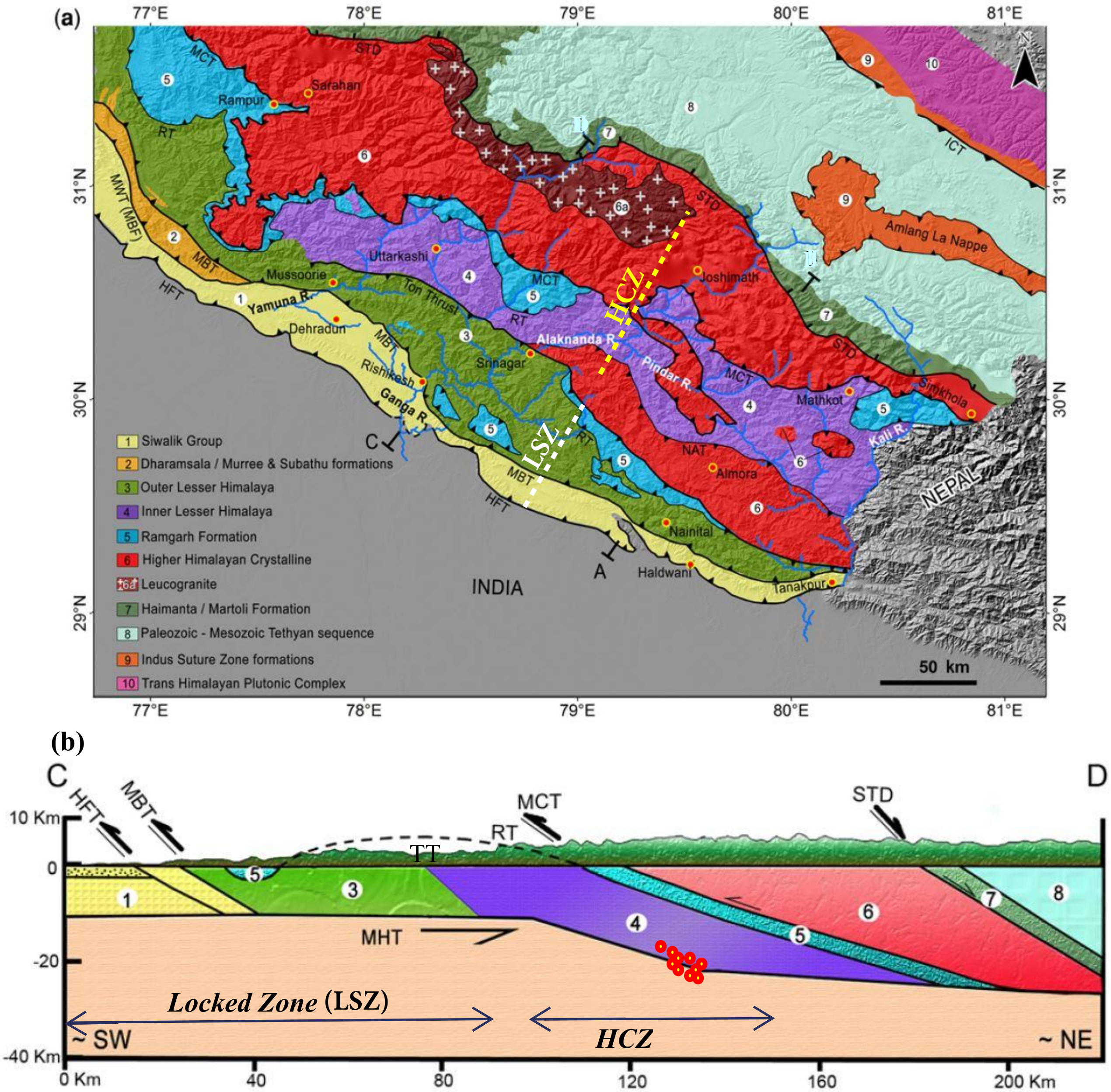

Supply. Fig. 1: (a), Geological map of the Garhwal-Kumaun Himalaya (after Thakur, V. C., et al 2018). High Compressional Zone (HCZ) and the Low Strain-rate Zone (LSZ) are marked in yellow and white dotted lines respectively. (b), 2D cross-section of the profile. The locked zone and the High compressional zone are marked and the red dots indicate the seismicity clustering in the downdip of MCR.

(a) Magnitude vs Time (Above 100 nstrain/a zone)

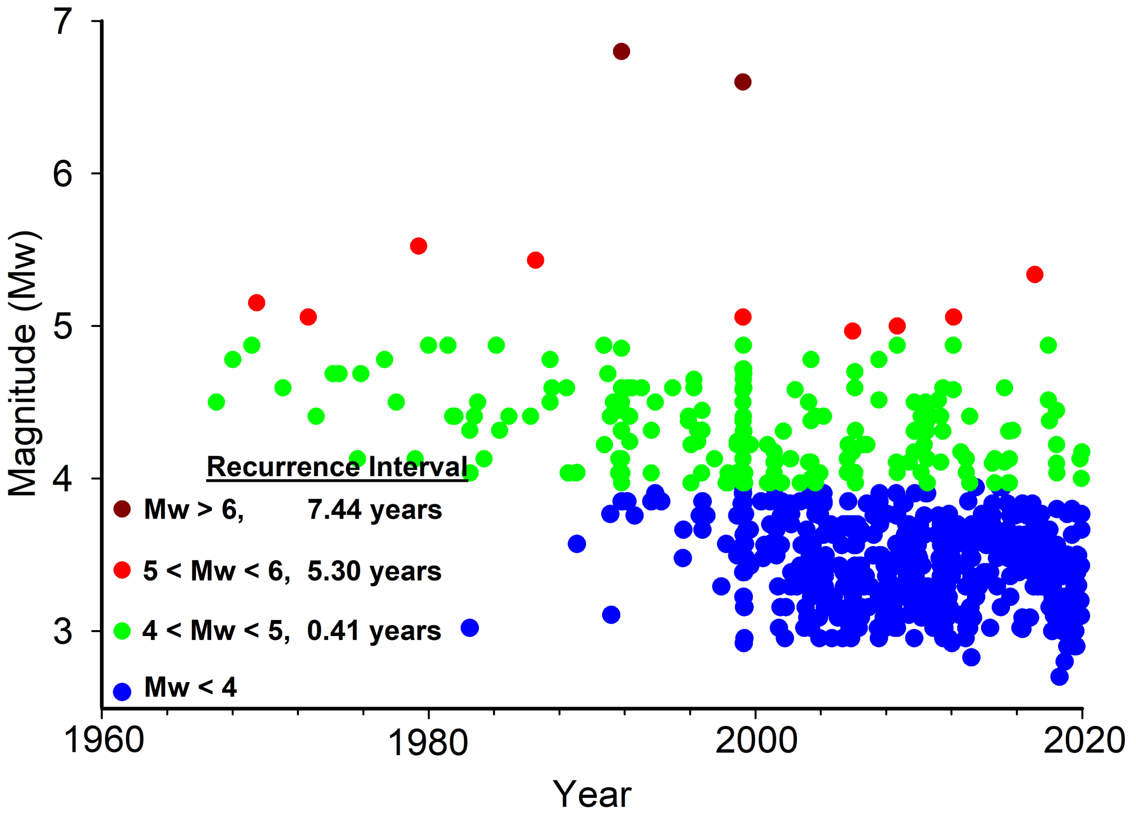

(b) Magnitude vs Time (Below 100 nstrain/a zone)

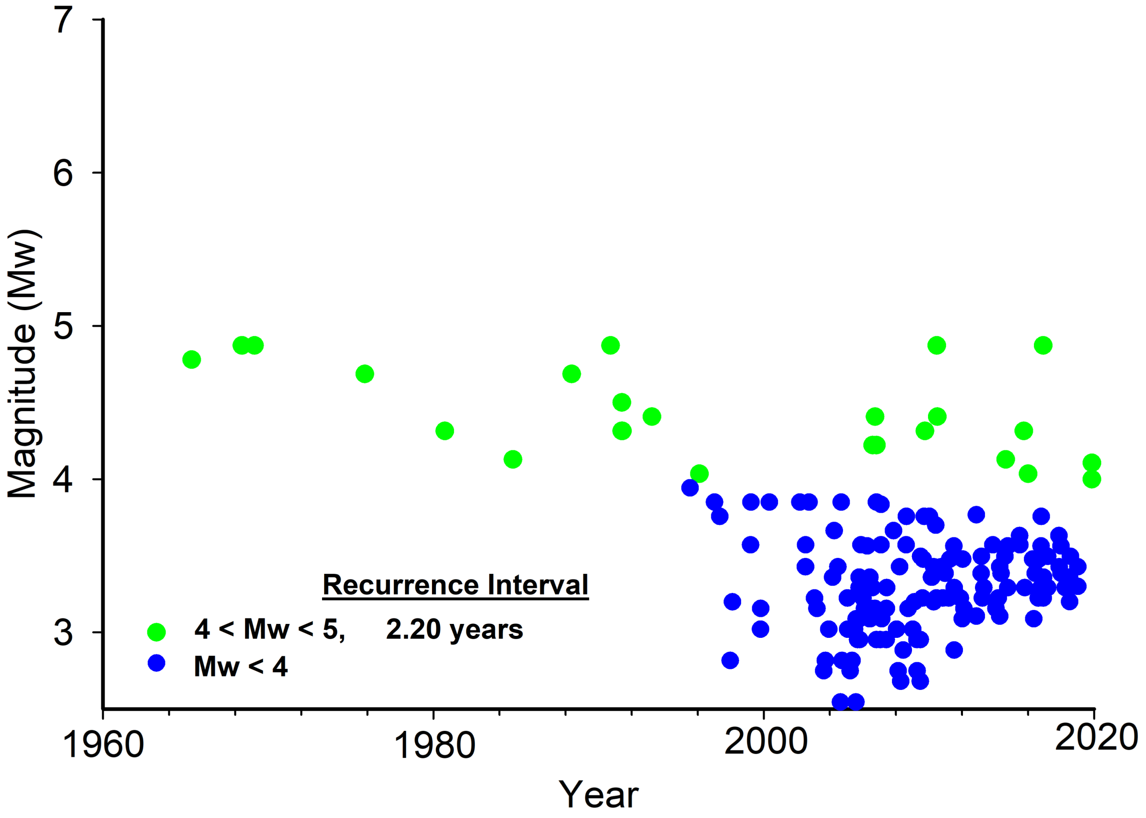

Supply. Fig. 2: Magnitude vs Time plot (a) above 100 nstrain/a zone and (b) below 100 nstrain/a zone.

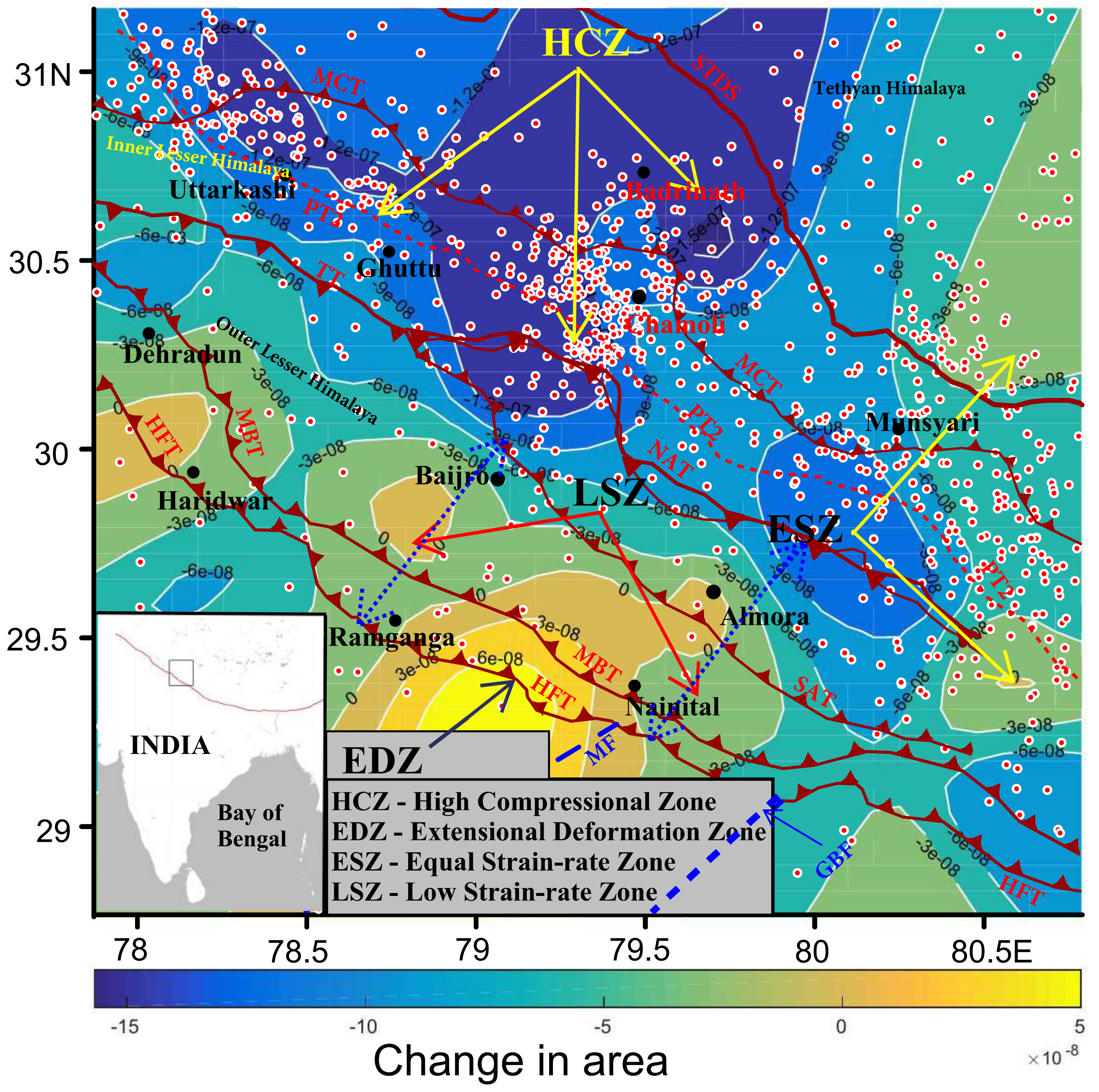

HCZ
LSZ
ESZ
EDZ
Tethyan Himalaya
Inner Lesser Himalaya
Outer Lesser Himalaya
Uttarkashi
Badrinath
Ghuttu
Chamoli
Dehradun
Munsyari
Haridwar
Baijro
Almora
Ramganga
Nainital
MCT
STDS
PT2
TT
MBT
HFT
NAT
SAT
MF
GBF
INDIA
Bay of Bengal
HCZ - High Compressional Zone
EDZ - Extensional Deformation Zone
ESZ - Equal Strain-rate Zone
LSZ - Low Strain-rate Zone
31N
30.5
30
29.5
29
78
78.5
79
79.5
80
80.5E
-15
-10
-5
0
5
× 10^-8
Change in area

Supply. Fig. 3: Figure represents the rate of change of surface area in the region. Colored contour represents the change in area rates. Seismicity and major tectonic features in the region are marked in the contour map. High compression zone (HCZ), Extensional deformational zone (EDZ), Equal strain-rate zone (ESZ), Low strain-rate zone (LSZ), and the Locked zone (Blue dotted double headed arrows) are marked. The base map is generated using the grid strain software (Teza et al., 2008) and further modifications are done using the grapher software. (https://www.goldensoftware.com/products/grapher).

Supplementary Note: The rate of surface area changes also supporting the compressional and extensional zones of strain in the region (supply. Fig. 3). In the figure, positive eigenvalues represent the extension whereas the negative values indicate compression. The extensional zone in the proximity of the Moradabad fault shows the positive eigenvalue contours ($6x10^{-08}$/year) and whereas all other regions show negative eigenvalues. The negative eigenvalues increases towards the high compressional zone in the MCT and a closed contour of maximum value $-1.5x10^{-07}$/year is seen in the Badrinath region.

Longitude	Latitude	Depth	Strike 1	Dip 1	Rake 1	Strike 2	Dip 2	Rake 2
81.092	29.598	16	102	65	97	266	26	75
80.95	29.42	22.3	108	69	89	290	21	91
78.4748	31.8146	17.7	58.03	51.89	3.11	326.11	87.55	141.85
78.4741	31.8466	21.7	56.41	34.53	-101.64	250.45	56.28	-82.09
78.19	30.48	15	152	80	104	278	17	37
78.7907	30.756	16.1	85.14	65.22	43.77	333.27	51.09	147.41
78.774	30.78	19	18	38	172	115	85	52
78.24	30.22	15	112	78	84	317	14	115
80.6116	29.8171	24.2	79.09	73.68	103.97	217.55	21.36	50.48
80.6116	29.8171	24.2	133.17	59.43	115.17	270.44	38.81	54.25
80.532	29.845	14	101	75	112	225	26	37
80.29	29.43	15	122	73	97	279	19	68
79.3354	30.3635	23.8	93.75	75.95	102.18	232.12	18.51	49.87
79.403	30.512	15	107	77	90	287	13	90
79.21	30.38	15	115	83	92	280	7	75
79.288	30.2994	25.6	18.64	69.84	152.05	119	63.89	22.57
81.154	31.024	10.1	140	44	-107	343	49	-74
80.97	30.88	12	17	76	-17	112	73	-165
78.83	30.12	34.2	117	67	92	293	23	86
80.31	30.06	30.7	41	59	-43	156	54	-141
80.24	30.08	22.9	234	75	-32	333	59	-163
82	30.53	22.5	202	76	-19	297	71	-165
80.33	29.55	14.1	85	74	71	318	25	140
80.71	29.43	18.8	96	67	70	318	30	128
80.754	29.698	26.1	98	46	69	307	48	111
80.754	29.698	26.1	98	46	69	307	48	111
78.2	31.2	33	277.46	48.54	121.43	54.93	50.25	59.46
82.7188	32.3928	27.1	33.88	82.96	-27.22	127.48	63	-172.1
82.92	32.32	20.9	118	70	172	210	83	20
80.38	29.57	34	110	67	79	315	26	113
79.0787	30.5773	14.6	95.62	73.25	50.48	346.37	42.38	154.69
79.1645	30.6544	16.1	103.25	59.13	92.05	279.27	30.93	86.58
78.85	30.41	20.6	102	68	85	295	23	102

Supply. Table. 1: Available focal mechanism solutions of significant earthquakes in the Garhwal-Kumaun region. Focal mechanism solutions are collected from Global CMT, ISC Focal Mechanism.
